# A Soft Pneumatic Inchworm Double balloon (SPID) for colonoscopy

**DOI:** 10.1038/s41598-019-47320-3

**Published:** 2019-07-31

**Authors:** Luigi Manfredi, Elisabetta Capoccia, Gastone Ciuti, Alfred Cuschieri

**Affiliations:** 1 Institute for Medical Science and Technology (IMSaT), Division of Imaging and Technology, School of Medicine, University of Dundee, Dundee, DD2 1FD UK; 2 The BioRobotics Institute, Scuola Superiore Sant’Anna, 56025 Pisa, Italy

**Keywords:** Physical examination, Biomedical engineering, Polymers, Mechanical engineering

## Abstract

The design of a smart robot for colonoscopy is challenging because of the limited available space, slippery internal surfaces, and tortuous 3D shape of the human colon. Locomotion forces applied by an endoscopic robot may damage the colonic wall and/or cause pain and discomfort to patients. This study reports a Soft Pneumatic Inchworm Double balloon (SPID) mini-robot for colonoscopy consisting of two balloons connected by a 3 degrees of freedom soft pneumatic actuator. SPID has an external diameter of 18 mm, a total length of 60 mm, and weighs 10 g. The balloons provide anchorage into the colonic wall for a bio-inspired inchworm locomotion. The proposed design reduces the pressure applied to the colonic wall and consequently pain and discomfort during the procedure. The mini-robot has been tested in a deformable plastic colon phantom of similar shape and dimensions to the human anatomy, exhibiting efficient locomotion by its ability to deform and negotiate flexures and bends. The mini-robot is made of elastomer and constructed from 3D printed components, hence with low production costs essential for a disposable device.

## Introduction

Colorectal cancer (CRC) is the third most common cause of cancer worldwide^1^. Regular screening of the asymptomatic population can drastically reduce the mortality rate (5-year survival rate above 90% in case of early, stage I, diagnosis)^2^. CRC screening involves several procedures^3,4^, although the gold standard remains optical colonoscopy^5^ because of its high sensitivity in detecting small or sessile polyps^2^ and low false negative rate^6^. Colonoscopy is performed with a flexible 1.6 meter long colonoscope by a trained colonoscopist. A colonoscope is a semi-flexible tube with 2 cable-driven degrees of freedom (DOFs) in the tip (pitch and yaw angles), which are controlled by an external manual handle. Two additional DOFs are provided by rotating the colonoscope through its handle and by pushing the instrument through the anus. This pushing force is required to introduce and navigate the colonoscope inside of the lumen of the colon to reach the caecum (top expanded section). During this procedure, the long and passive tube is pushed against the colonic wall, inducing pain and discomfort for the patient^7,8^. For this reason, colonoscopy for screening and diagnosis of symptomatic diseases may often require sedation and analgesia. Aside from discomfort, rare instances of colonic perforation^9^ requiring emergency surgery to prevent peritonitis remain a concern. Cleaning and chemical sterilization of colonoscopes are necessary as the device is expensive, non-disposable and requires periodic servicing and maintenance to ensure a continued optimal function.

The advantage in using a mini-robot to carry out a colonoscopy is that once inserted through the anus, the device will travel by its intrinsic locomotion capability to the caecum virtually abolishing pain and discomfort, as it avoids pressure on the colonic wall and mesenteric tending by loop formation. In addition, standard conventional optical push colonoscopy is a difficult procedure requiring a long period of training for acquisition of the required level of proficiency for safe expert execution and interpretation^10,11^. Robotic colonoscopy dispenses with this long-proficiency-gain curve, averaging 2–3 years to attain competent and safe caecal intubation. This mini-robot should include a camera in its distal end for visual inspection and instruments for treatment. A soft-tether is necessary for high quality video transmission to an external user console, powering and control of the robot. The tether provides also a safety mechanism for withdrawing the mini-robot in case of malfunction. The friction provided by the tether against the colonic wall is a drag force in the locomotion. To overcome this force a robot needs to provide enough locomotive traction, although this can be challenging considering the small size, light weight of a mini-robot and the slippery colonic mucosa. The more conventional approach to design robots for colonoscopy is essentially by construction of components made of rigid miniaturized mechanical parts^12–19^, which may require expensive high precision machining. Thus, such a mini-rigid robotic colonoscope must be re-usable so that the device will be a financially viable proposition. Even if fully developed, it is most unlikely that it would reduce significantly the costs of screening colonoscopy for colorectal cancer.

The use of soft materials has the advantage of reducing the forces applied to the colonic wall and consequently diminishing pain and discomfort to the patient during the procedure. Because of the low mechanical stiffness, a soft robot can perform dexterous movements and follows the 3D-shape contours of the colonic lumen without the need of a complex active closed-loop control^20^. An additional advantage of using soft materials is the low production costs^21^. The body of the robot can be produced by “injection moulding” at a very low cost^22^, enabling the robot to be marked as a disposable device and thus avoiding the issues of cleaning, disinfection, and maintenance. This will drastically reduce the health care costs compared to the traditional optical colonoscopy and also the overall acceptability by patients.

Design of a soft mini robot for colonoscopy is challenging because of the limited lumen available of the colon, with an internal diameter ranging from 40 to 80 mm^23,24^, as well as the locomotion challenge rendered difficult by the device having to successfully negotiate the colonic flexures. The design should also provide space to locate a camera at the distal-end for visual inspection, introduction and use of therapeutic instruments. When the size of a soft robot is scaled down inclusion of all these requirements in the design may become challenging because of the high air activation pressure^25^ or high activation voltage^26,27^ needed. In addition, these have to meet stringent medical regulatory standards. Micro and nanorobots, developed during the last decade, have lacked therapeutic and diagnostic functionalities. Several challenges have to be resolved before untethered robots can be realised for medical use^28^.

Soft robots can be actuated by shape memory alloys^29,30^, cables and air^31–35^, external magnetic field^36^, light^37^, or by a combination of different actuation systems^38^. Nature is inspirational for such designs^39,40^. Inchworm locomotion led to several reported studies^41–45^ together with anchoring methods included in the design to increase the contact force and to address the direction of the locomotion. Toroidal balloons have been proposed to anchor the inchworm device inside a rigid tube^46^ or a colon^18^. However, all the previous balloon inchworm-like locomotion designs rely on a linear actuator with 1 DOF that can be bent passively by pushing against the luminal internal wall to proceed around corners^18,46^. This, however, may not be possible when the device has to negotiate acute corners or exceed high force against the inner wall of a lumen to achieve passive bending. The balloon design reported in previous studies^18,46^ has limited variation of the external diameter reducing the anchorage force possible in the various regions of the colon.

**Figure 1:**
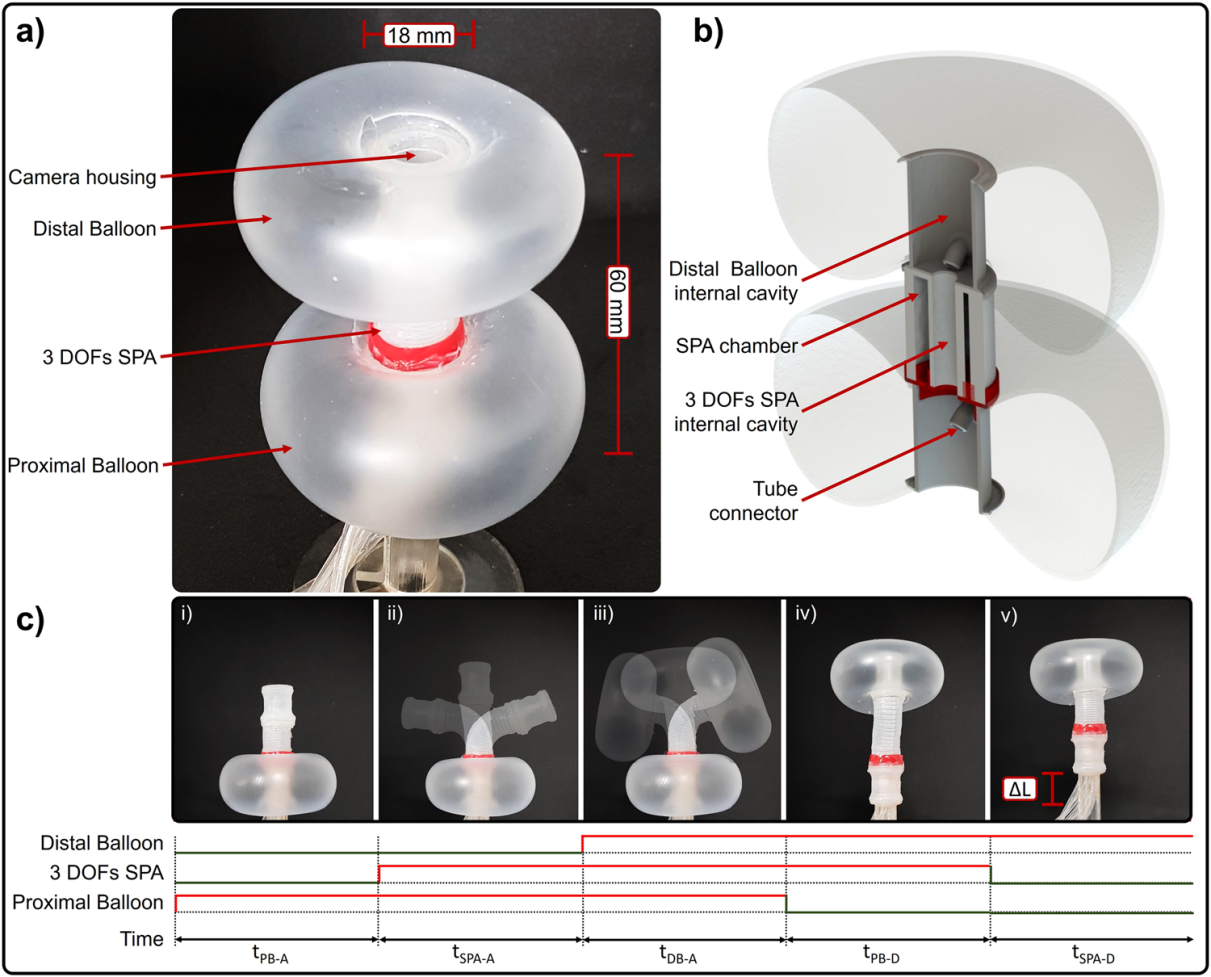
SPID design: (**a**) perspective view with the distal and proximal balloon activated; (**b**) cross-sectional showing the available inner space of the balloons and the SPA; (**c**) five steps bio-inspired locomotion, where (i) *t*_*PB*–*A*_ is the time needed to activate the proximal balloon, (ii) *t*_*SPA*–*A*_ is the time to activate the SPA in line with the orientation of the colonic lumen, (iii) *t*_*DB*–*A*_ is the activation time of the distal balloon providing anchorage, (iv) *t*_*PB*–*D*_ is the deactivation time of the proximal balloon, and (v) *t*_*SPA*–*D*_ is the deactivation time of the SPA to move it forward.

In the present study we propose a Soft Pneumatic Inchworm Double balloon (SPID) mini-robot consisting of two balloons connected by a 3 DOFs soft pneumatic actuator (SPA), with a total 5 DOFs as shown in Fig. 1a. Both balloons have a cylindrical frame with an inner cavity of 13 × 20 mm (Fig. 1b). An additional advantage of using balloons is the possibility of increasing the contact force despite the light weight of the mini-robot. The central pneumatic actuator can extend (1 DOF) and rotate around 2 axes (2 DOFs) to move forward with enough dexterity to go around narrow acute corners, including colonic flexures. The inchworm locomotion entails 5 sequential steps as shown in Fig. 1c: (i) proximal balloon activation to anchor the mini-robot within the circular colonic wall; (ii) extension of the SPA forwards following the contour and orientation of the colonic lumen; (iii) activation of the distal balloon to provide the required anchorage; (iv) deactivation of the proximal balloon and (v) contraction of the SPA to move forwards. The design parameters of SPID, i.e., range of motion, external diameter, and overall length were chosen to conform with the average size and shape of a human colon. Advantages of this design compared to the previous reported devices are (i) high dexterity in locomotion with 5 DOFs to overcome narrow bends and flexures, and (ii) the novel balloon design for improved anchorage of the mini-robot in different sections exerting a low external pressure in the colonic wall, (iii) whilst providing enough inner space for housing camera, electronics, cables and tubes, required for endoscopic examination.

## Results

It is well established that one of the main challenges for a robotic colonoscopy consists of the ability of the device to negotiate the acute angled splenic flexure at the junction between the transverse and the descending colon. This flexure has an angle ranging between 40° to 50° and a diameter of 30 mm after *CO*_2_ inflation of the colon^24^.

**Figure 2:**
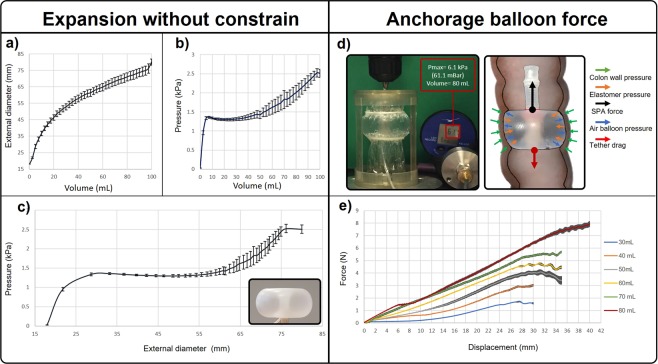
Experiment results of the balloon with radial expansion reporting external diameter vs. air volume (**a**), activation air pressure vs. air volume (**b**), activation air pressure vs. external balloon diameter (**c**). Static forces and pressures of the balloon inside the colon wall involved resulting from the inchworm locomotion are shown in the top-right section of sub-fig. (**d**). Sub-figure (**e**) shows maximal anchorage force that increased with the volume of air exerting 7.9 N with internal pressure of 6.1 kPa.

The two balloons proposed in SPID design are made from Ecoflex 00–30 (Smooth-on-Inc., PA, USA) and can be activated with a low air pressure ensuring low pressure exerted on the colonic wall. The experiments were performed in a quasi-static configuration activating the balloon with air controlled manually by using an external piston-cylinder and measuring the inflated volume and air pressure. Each experiment was performed 5 times with mean value and standard deviation shown in the graphs. Each balloon has an external diameter at rest of 18 mm and when activated it expands above 80 mm (Fig. 2a), with an activation pressure below 2.5 kPa without external constrain (Fig. 2b). The external diameter vs. air volume exhibits a monotonic function, resulting in an air volume as a feedback-reference for the implementation of a closed-loop control; i.e., by controlling the piston stroke in a pneumatic cylinder. The pressure vs. air volume graph exhibits 3 phases. In phase one, as the air volume rises from 0 to 10 mL, the pressure increases rapidly to 1.4 kPa with a negligible increase of the balloon external diameter. In phase two, as the air volume rises from 10 to 50 mL, the pressure decreases slightly with its value about 1.4 kPa. In this phase, the balloon diameter starts to increase. Phase three, when the air volume exceeds 50 mL of air volume, the pressure increases with a linear fashion up to 2.5 kPa.

The graph reported in Fig. 2c is obtained by combining the two graphs from Fig. 2a,b to relate the external diameter of the balloon with the internal air pressure. This graph shows a non-monotonic function. This implies that there is no constant relation between a given diameter and a given pressure. This behaviour is explained by the fact that the internal air pressure increases the wall tension as the diameter increases (Laplace’s law) in accordance with the mechanical properties of Ecoflex. The characteristics of this balloon require that, to adjust its diameter, a closed-loop control system has to use the air volume as a feedback-reference input, because a given balloon external diameter cannot be controlled by using the air pressure.

The inflated balloon wide diameter allows the robot to secure a stable anchorage inside the colon between haustra providing sufficient force to move it forward. The anchorage force was measured by using an Instron 5564 dual column with the load cell connected to the balloon (Fig. 2d) and by pulling it up at a constant speed of 1 mm/s. The force profile vs. displacement for each volume of air inflated in the balloon is shown in Fig. 2e. The maximal anchorage force was 7.9 N with a balloon activation air volume of 80 mL and pressure of 6.1 kPa. The force was tested by using a vertical tube made from transparent thin plastic film (15 *μm* thickness) with the balloon being anchored between 2 elastic bands simulating a haustral fold.

**Figure 3:**
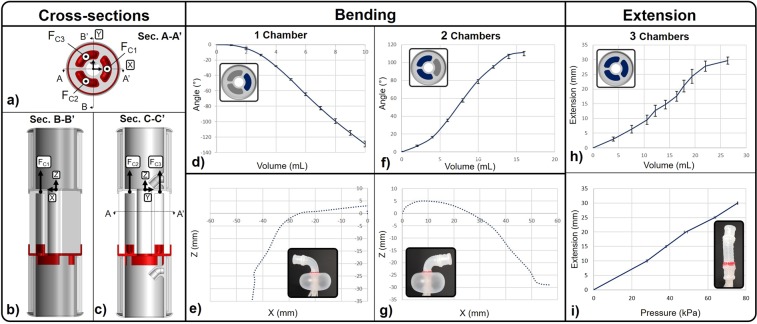
Sub-fig. (**a**) shows cross-section of the SPA with 3 chambers, *C*_1_, *C*_2_, *C*_3_, and their forces, *F*_*C*1_, *F*_*C*2_, *F*_*C*3_. Cross-section *A* − *A*′, (**b**), shows the force produced by 1 chamber, *C*_1_, during negative bending. Cross-section *B* − *B*′, (**c**), shows the activation of the 2 chambers during positive bending. Experiments of the SPA unrestricted activation when 1 chamber (**d**,**e**) and 2 chambers (**f**,**g**) are activated. Sub-fig. (**d**,**f**) show the bending angle and the cross-section with the chamber activated in blue. Sub-fig. (**e**,**g**) show the position of the tip in the X-Z plane. Sub-fig. (**h**) shows the vertical extension when 3 chambers are activated and the air pressure thereof in sub-fig. (**i**).

Free displacement studies of SPID were performed by activating the SPA. Its design consists of 3 chambers (*C*_1_, *C*_2_, *C*_3_) symmetrically disposed around the central cavity as shown in the cross-section *A* − *A*′ in Fig. 3a. Figure 3b shows the cross-section *B* − *B*′ and the force produced by the activation of chamber *C*_1_ for negative bending. Figure 3c shows the cross-section *C* − *C*′ and the force produced by the activation of chambers *C*_2_, *C*_3_ for positive bending. Experiments of the negative bending around the Y axis are shown in Fig. 3d with a maximal value of −130°. The activation of the chamber *C*_1_ moves the distal part of SPID in the X-Z plane, with the trajectory reported in Fig. 3e. Experiments of the positive bending around the Y axis are shown in Fig. 3f with a maximal value of 110°. The trajectory of the bending in the plane X-Z is reported in Fig. 3g. Figure 3e,g show similar bending trajectories. However, the higher force generated by the simultaneous activation of 2 chambers during the positive bending produces an X displacement wider than the negative bending, when only 1 chamber is activated. The extension along the Z axis is obtained by the simultaneous activation of 3 chambers *C*_1_, *C*_2_, *C*_3_ producing a maximal extension of 30 mm as shown in Fig. 3h. The SPA extension vs. air pressure monotonic function is shown in Fig. 3i. This monotonic behaviour is different from the non-monotonic balloon profile and is due to the anisotropic structure of the SPA design, the wall of which is reinforced with cotton threads to constrain lateral expansion. Details of the construction process are reported in the Supplementary Material. During its activation, the chambers’ cross-section area remains almost constant with a generated force proportional to the internal air pressure.

**Figure 4:**
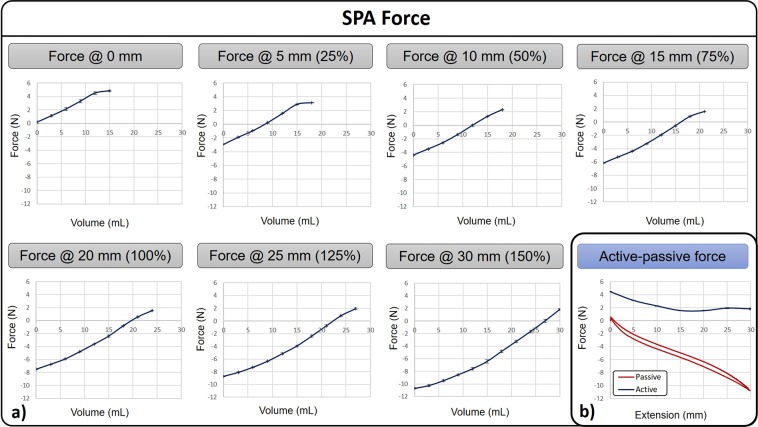
Sub-fig. (**a**) shows experiments of the SPA activation force with its extension up to 30 mm. Sub-fig. (**b**) shows the force produced when the SPA is activated, blue line, and when deactivated, red line.

Figure 4 shows blocking force of SPID. These experiments were performed by using an Instron 5564 dual column and blocking SPID from the base to the load cell. Graphs force vs. air volume are shown in different extension from 0 up to 30 mm (Fig. 4a) with a maximal pushing longitudinal force of 4.8 N. This force is needed to advance SPID forward and open its path in case the luminal collapse. The output force decreases with the extension due to the increase of the resistance of the polymer when it is stretched. Figure 4b shows the force vs. extension profile when the SPA is activated (blue line), and the passive force produced by the polymer mechanical resistance when the SPA is deactivated (red line). The active force decreases linearly from 0 to 15 mm and then retains a value around 2 N. The passive force has an almost linear profile with a negligible hysteresis due to the mechanical properties of the polymer. The passive force is required to pull the tether and overcome its drag during the locomotion. These studies reported high dexterity and high force vs. weight ratio.

The tether to activate the two balloons and the SPA is composed of 5 silicon tubes with an external diameter of 2.6 mm each for the SPA, and 3 mm each for the balloons.

**Figure 5:**
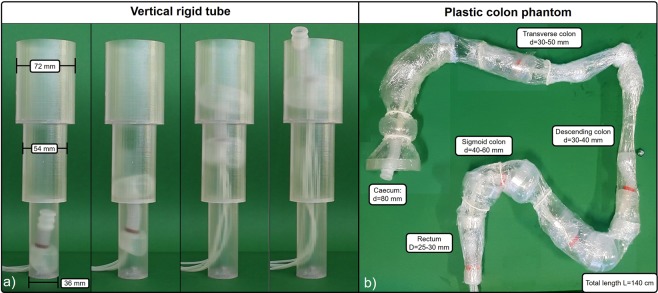
Experiments of SPID in a vertical tube (**a**), showing the ability of the two balloons to adapt their diameters to different sections. Sub-fig. (**b**) shows SPID in different sections of the plastic colon. Videos of these experiments are included in the Supplementary Material V1 and V2.

Vertical locomotion and its capability to adapt the shape to different colonic sections were tested in a rigid vertical tube with internal diameters of 36 mm, 54 mm, and 72 mm, respectively, each with a length of 100 mm, providing a total of 300 mm, as it is shown in Fig. 5a and the Supplementary Video V1. This experiment shows the ability of the balloons to conform to the shape of different colonic diameters.

A colon phantom was constructed to conform with the shape and size of the average human colon^24^ and was used to investigate the locomotion performance of SPID, as it is shown in Fig. 5b and the Supplementary Video V2. This set up addressed the locomotion in a more challenging environment including the 3D path as well as thin and weak plastic wall. Lubricant (Vaseline, Unilever, London, UK) was applied between the mini-robot and the wall made of transparent thin plastic film to reduce the locomotion friction. Elastic bands were used to simulate haustral folds in the colon. SPID showed high dexterity in negotiating narrow corners with good locomotion speed. The experiments were performed in accordance with a standard colonoscopy procedure. Hence, in the initial phase of the colonoscopy procedure, the endoscopist is focused on reaching the caecum. Thereafter, inspection is undertaken during the second stage as the endoscope is withdrawn slowly. SPID experiments reproduced the same procedure, showing that was able to reach the caecum in 8′30″ covering a total length of 1.4 m at an average speed of 2.8 mm/s. After caecal intubation, the mini-robot was withdrawn by traction on the air tubes. This phase took about 1′ with an average speed of 25 mm/s although in a real scenario this may take longer for a precise evaluation of the colonic mucosa. During this phase SPID could be activated, enabling forward locomotion for subsequent inspection when considered necessary to ensure that no abnormality was missed. The balloon, when anchored, allows a precise control and steering of the mini-robot tip for inspection and/or treatment.

## Discussion

This study reports a soft pneumatic inchworm double balloon, refereed to SPID, for colonoscopy. The mini-robot is made of 3D printed components and Ecoflex™ 00–30 with high compliance and flexibility for intrinsic locomotion demonstrated by experiments in a plastic deformable colon phantom. The dexterous and compliant behaviour of the 3 DOFs soft pneumatic actuator connecting the 2 balloons enables the mini-robot to negotiate acute corners and the highly compliant double balloon structure readily adapt to conform with shape of the various colonic sections and their diameters. Construction from soft materials carries several physical advantages including innate flexibility, gentle atraumatic contact with the colonic wall and low production cost, essential for production of a disposable device. This can avoid issues concerning sterilisation, cross contamination and maintenance. The low Young’s modulus provides a passive compliant interaction with the colonic wall reducing pressure for anchorage. During the experiments, the balloons have never sustained any air leakage, essential for assuring the device reliability. A camera is needed for the colon inspection, which can be mounted in the cavity of the distal balloon. Traditional biopsy instruments can be used both for diagnostic and therapeutic functions. SPID can incorporate a tube extending from the camera to an external console for insertion of instruments for treatment of suspect lesions.

The present study was primarily concerned with the locomotion design and its transit rate in a plastic colon phantom. The insertion of the tether through the anus will produce additional friction and drag force not considered in the present study. However, this friction can be almost abolished by use of an external device to feed the tether during the locomotion or with the use of a dedicate access port. The activation of the SPA chambers and the balloons was obtained by using manual controlled external pistons-cylinders, successfully demonstrating its functionalities. An active control will improve the locomotion speed, manoeuvrability, and will ease the procedure by reducing activation and deactivation times. Such control can be implemented via an external user console with a joystick or by using a smart-control to follow autonomously the colonic lumen. This should ensure very precise control of the mini-robot as well as assistance in the training or in the use of SPID by a technician or nurse practitioner. In such scenario, a trained colonoscopist would potentially supervise several simultaneous ongoing procedures acting, e.g., requesting biopsy or reverse locomotion to obtain a second look, with a disruptive reduction of cost. Finally, SPID by reducing the pressure against the colon wall, may contribute to increase the compliance rate for CRC screening of the asymptomatic population, towards mass screening campaigns for early diagnosis.

## Methods

The basic concept of SPID involves the design of the double balloon and the SPA. Each part was constructed and tested before the assembly to avoid any issue in the final functional outcomes. A colonic phantom was designed and constructed to test the locomotion performance based on the size and configuration of the average human colon anatomy. The following sections report on the design details of each component.

### Balloon for anchorage

Previous studies proposed balloon designs with a limited range in expansion of the external diameter, and lack on internal space to accommodate the image system^18,46^, despite this requirement is essential for the design of a mini-robot for colonoscopy. A large range in the external diameter (on inflation), allows the robot to be anchored in the different sections of the colonic wall. An internal cavity is necessary for accommodation of the electronic components for the control, i.e., IMU (inertial measurement unit) or camera. Both requirements have been addressed in the design of the balloons.

The balloon structure includes a 3D printed cylindrical frame made of Vero-Clear surrounded by a thin layer (thickness of 0.6 mm) of silicon rubber Ecoflex™ 00–30. Compared to other silicon rubbers, Ecoflex™ 00–30 is the one that exhibits lower shore hardness and higher elongation break. This results in a lower activation pressure and higher compliant behaviour, essential to reduce forces applied to the colonic wall and consequently increasing patient compliance. A higher stiffer elastomer would require higher activation pressure, which may cause problems in case of balloon rupture. A tube connector is located in the inner part of the frame for the balloon activation. The construction process is described in detail in the Supplementary Materials.

The balloon, when activated, adapts its shape to the colonic haustral fold producing an anchorage force (*F*_*A*_) essential for the inchworm locomotion. This force has 2 major components: Coulomb friction (*F*_*C*_) and marginal resistance (*F*_*M*_). The Coulomb friction is related to the force of the balloon against the colonic wall (*F*_*B*_) and the coefficient of friction *μ*. This force includes also the weight of the robot (*F*_*R*_) although, because of its light weight (10 g), this force is negligible compared to *F*_*B*_. The *F*_*C*_ can be low because of the slippery colonic mucosa surface. The marginal resistance is related to the longitudinal deformation of the colonic wall.

During a colonoscopy, air or gas is used to inflate and expand the colonic lumen with an internal pressure up to 7.6 kPa^47,48^, which is higher than the balloon activation pressure required in SPID. The pressure of the balloon against the colonic wall is the result of the internal air pressure (*P*_*B*_) minus the elastomer mechanical pressure (*P*_*E*_). This produces an external pressure on the colon wall, which is much lower than the pressure pain threshold encountered during standard colonoscopy.

### SPA design

The two balloons are connected with a 3 DOFs soft pneumatic actuator (SPA). This design relies on a positive differential pressure (PDP) rather than a buckling actuator^49^, that relies on negative differential pressure (NDP). PDP actuators can provide a positive force to extend and pushing the distal balloon forward. This represents a considerable advantage for a colonoscopic mini-robot as it enables the device to open a collapsed colonic lumen. A pushing active force improves the ability of SPID to negotiate round corners adapting its shape by its intrinsic flexibility to conform with the contours of the flexure. The realisation of a PDP actuator with 3 DOFs imparts a further advantage by being considerably smaller than a NDP because of its simpler manufacturing process. When deactivated, a PDP actuator relies on a passive force provided by the mechanical properties of the elastomer as it contracts and pulls forward the proximal balloon. The main limitation in using a NDP actuator is the manufacture of 3 DOFs with an inner cavity in a small and compact design. Because of the established advantages of a PDP vs. a NDP actuator, the proposed robot design includes a PDP soft pneumatic actuator.

The SPA is composed of 3 longitudinal chambers and a central 5 mm circular cavity, with a cross-section area of 2.6 *mm*^2^. The external diameter is 18 mm and a total length of 20 mm. Several lengths vs. diameter ratios have been tested. The one selected for SPID exhibited a good compromise between lateral bending enabling high dexterity, and longitudinal elongation to enhance locomotion speed. Higher dexterity could have been achieved by using 4 chambers although 1 more tube would have increased the tether diameter and hence the drag force with inevitable reduction of the locomotion speed. Supplementary Material describes the construction process.

### Colon phantom

Colonoscopy robots have been tested *ex vivo*^7,50^, in synthetic phantoms^18,48,51,52^, and *in vivo* in pigs^18^. An *ex vivo* pig colon mounted on a work bench lacks muscle tone and is flaccid. Besides, haustral folds are reduced in the *ex vivo* colon specimen. This is on sharp contrast to the normal *in situ* living human colon, which exhibits more than 600 haustra along its length^53^. In addition, an *ex vivo* pig colon is floppy and collapsed, significantly different from a live human colon where air or *CO*_2_ are used to expand the colonic lumen during colonoscopy. A live pig has similar mechanical properties, although its spiral colonic configuration is different from the shape and configuration of the human colon. An available off-the-shelf phantom^54^ has rubber mechanical properties that differ considerably from the consistency of the human colon. This will thus exert high force against a robot capable of intrinsic locomotion.

In our experiments, to overcome all these issues, a colon phantom was constructed specifically to test locomotion. This has a total length of 1.4 m and a diameter ranging from 25 mm up to 80 mm. Annular 3D printed rings define the shape of the internal diameter of the colon. The colonic wall was made of transparent plastic films (15 *μm* wall thickenss). The low mechanical stiffness of the plastic film was needed in view of testing the effect of low pressures applied to the colonic wall. The entire length of the internal wall of the plastic phantom was lubricated with Vaseline to reduce the contact friction. Plastic bands were used to simulate haustral indentations. Details of the colon phantom are reported in the Supplementary Material.

## Supplementary information

Supplementary materials

Supplementary video 1

Supplementary video 2

## References

[1] Bray F (2018). Global cancer statistics 2018: Globocan estimates of incidence and mortality worldwide for 36 cancers in 185 countries. CA: a cancer journal for clinicians.

[2] Hamashima C, Shabana M, Okada K, Okamoto M, Osaki Y (2015). Mortality reduction from gastric cancer by endoscopic and radiographic screening. Cancer science.

[3] Bevan R, Rutter M (2018). Colorectal cancer screening—who, how, and when?. Clin Endosc.

[4] Schreuders EH, Grobbee EJ, Spaander MCW, Kuipers EJ (2016). Advances in fecal tests for colorectal cancer screening. Current treatment options in gastroenterology.

[5] Dekker E, Rex DK (2018). Advances in crc prevention: Screening and surveillance. Gastroenterology.

[6] Schreuders EH (2015). Colorectal cancer screening: a global overview of existing programmes. Gut.

[7] Valdastri P, Simi M, Webster RJ (2012). Advanced technologies for gastrointestinal endoscopy. Annual Review of Biomedical Engineering.

[8] Appleyard MN (2000). The measurement of forces exerted during colonoscopy. Gastrointestinal Endoscopy.

[9] Polter, D. E. Risk of colon perforation during colonoscopy at baylor university medical center. In *Baylor University Medical Center Proceedings*, vol. 28, 3–6 (Taylor & Francis, 2015). 10.1080/08998280.2015.11929170 PMC4264696 25552784

[10] Jang HJ (2017). Training in endoscopy: Colonoscopy. Clinical endoscopy.

[11] Kim SY, Kim H-S, Park HJ (2019). Adverse events related to colonoscopy: Global trends and future challenges. World journal of gastroenterology.

[12] Cheung, E., Karagozler, M. E. & Sitti, M. A new endoscopic microcapsule robot using beetle inspired microfibrillar adhesives. In *Proceedings*, *2005 IEEE/ASME International Conference on Advanced Intelligent Mechatronics*, 551–557, 10.1109/AIM.2005.1511040 (2005).

[13] Gao J (2017). Locomotion enhancement of an inchworm-like capsule robot using long contact devices. The International Journal of Medical Robotics and Computer Assisted Surgery.

[14] Hosokawa D, Ishikawa T, Morikawa H, Imai Y, Yamaguchi T (2009). Development of a biologically inspired locomotion system for a capsule endoscope. The International Journal of Medical Robotics and Computer Assisted Surgery.

[15] Kim, B., Park, S., Yeol Jee, C. & Yoon, S.-J. An earthworm-like locomotive mechanism for capsule endoscopes. 2997–3002, 10.1109/IROS.2005.1545608 (2005).

[16] Breedveld, P. Development of a rolling stent endoscope. In *The First IEEE/RAS-EMBS International Conference on Biomedical Robotics and Biomechatronics*, *2006. BioRob 2006*, 921–926, 10.1109/BIOROB.2006.1639209 (2006).

[17] Maple JT (2013). Methods of luminal distention for colonoscopy. Gastrointestinal endoscopy.

[18] Wang, K., Ge, Y. & Jin, X. A micro soft robot using inner air transferring for colonoscopy. In *2013 IEEE International Conference on Robotics and Biomimetics (ROBIO)*, 1556–1561, 10.1109/ROBIO.2013.6739688 (2013).

[19] Valdastri P (2012). Magnetic air capsule robotic system: proof of concept of a novel approach for painless colonoscopy. Surgical Endoscopy.

[20] Marchese AD, Rus D (2016). Design, kinematics, and control of a soft spatial fluidic elastomer manipulator. The International Journal of Robotics Research.

[21] Franchetti M, Kress C (2017). An economic analysis comparing the cost feasibility of replacing injection molding processes with emerging additive manufacturing techniques. The International Journal of Advanced Manufacturing Technology.

[22] Isayev AI, Hieber CA (1980). Toward a viscoelastic modelling of the injection molding process. Rheol. Acta.

[23] Horton KM, Corl FM, Fishman EK (2000). Ct evaluation of the colon: Inflammatory disease. RadioGraphics.

[24] Alazmani A, Hood A, Jayne D, Neville A, Culmer P (2016). Quantitative assessment of colorectal morphology: Implications for robotic colonoscopy. Medical Engineering & Physics.

[25] Ranzani T, Gerboni G, Cianchetti M, Menciassi A (2015). A bioinspired soft manipulator for minimally invasive surgery. Bioinspiration & Biomimetics.

[26] O’Halloran A, O’Malley F, McHugh P (2008). A review on dielectric elastomer actuators, technology, applications, and challenges. Journal of Applied Physics.

[27] Cianchetti M, Mattoli V, Mazzolai B, Laschi C, Dario P (2009). A new design methodology of electrostrictive actuators for bio-inspired robotics. Sensors and Actuators B: Chemical.

[28] Sitti M (2018). Miniature soft robots — road to the clinic. Nature Reviews Materials.

[29] Rodrigue H, Wang W, Han M-W, Kim TJY, Ahn S-H (2017). An overview of shape memory alloy-coupled actuators and robots. Soft robotics.

[30] Le VH (2015). Shape memory alloy–based biopsy device for active locomotive intestinal capsule endoscope. Proceedings of the Institution of Mechanical Engineers, Part H: Journal of Engineering in Medicine.

[31] Manfredi, L., Yue, L., Zhang, J. & Cuschieri, A. A 4 DOFs variable stiffness soft module. In *IEEE RoboSoft*, 10.1109/ROBOSOFT.2018.8404903 (2018).

[32] Manfredi, L., Yue, L. & Cuschieri, A. A 3 DOFs mini variable stiffness soft pneumatic actuator. In *Proc*. *ACTUATOR 2018; 16th Int*. *Conf*. *New Actuators*, 1–4 (2018).

[33] Manfredi L, Putzu F, Guler S, Huan Y, Cuschieri A (2019). 4 DOFs hollow soft pneumatic actuator - HOSE. Materials Research Express.

[34] Marchese AD, Katzschmann RK, Rus D (2015). A recipe for soft fluidic elastomer robots. Soft Robotics.

[35] Tolley MT (2014). A resilient, untethered soft robot. Soft Robotics.

[36] Hu W, Lum GZ, Mastrangeli M, Sitti M (2018). Small-scale soft-bodied robot with multimodal locomotion. Nature.

[37] Huang C (2015). Miniaturized swimming soft robot with complex movement actuated and controlled by remote light signals. Scientific reports.

[38] Liu L, Towfighian S, Hila A (2015). A review of locomotion systems for capsule endoscopy. IEEE Reviews in Biomedical Engineering.

[39] Rus D, Tolley MT (2015). Design, fabrication and control of soft robots. Nature.

[40] Ricotti Leonardo, Trimmer Barry, Feinberg Adam W., Raman Ritu, Parker Kevin K., Bashir Rashid, Sitti Metin, Martel Sylvain, Dario Paolo, Menciassi Arianna (2017). Biohybrid actuators for robotics: A review of devices actuated by living cells. Science Robotics.

[41] Wang W (2014). Locomotion of inchworm-inspired robot made of smart soft composite (SSC). Bioinspiration & Biomimetics.

[42] Digumarti KM, Conn AT, Rossiter J (2018). umobot: replicating euglenoid movement in a soft robot. Journal of The Royal Society Interface.

[43] Lim J (2008). One pneumatic line based inchworm-like micro robot for half-inch pipe inspection. Mechatronics.

[44] Wang K (2017). Full-driving soft robotic colonoscope in compliant colon tissue. Journal of Medical Engineering & Technology.

[45] Alcaide, J. O., Pearson, L. & Rentschler, M. E. Design, modeling and control of a sma-actuated biomimetic robot with novel functional skin. In *2017 IEEE International Conference on Robotics and Automation (ICRA)*, 4338–4345, 10.1109/ICRA.2017.7989500 (2017).

[46] Verma MS, Ainla A, Yang D, Harburg D, Whitesides GM (2018). A soft tube-climbing robot. Soft robotics.

[47] Kozarek RA, Earnest DL, Silverstein ME, Smith RG (1980). Air-pressure-induced colon injury during diagnostic colonoscopy. Gastroenterology.

[48] Dehghani H (2015). Semi-autonomous locomotion for diagnostic endoscopy device 1. Journal of Medical Devices.

[49] Yang Dian, Verma Mohit S., So Ju-Hee, Mosadegh Bobak, Keplinger Christoph, Lee Benjamin, Khashai Fatemeh, Lossner Elton, Suo Zhigang, Whitesides George M. (2016). Buckling Pneumatic Linear Actuators Inspired by Muscle. Advanced Materials Technologies.

[50] Arezzo A (2013). Experimental assessment of a novel robotically-driven endoscopic capsule compared to traditional colonoscopy. Digestive and Liver Disease.

[51] Pourghodrat A (2014). Disposable fluidic self-propelling robot for colonoscopy. Journal of Medical Devices.

[52] Norton, J. *et al*. Rollerball: A mobile robot for intraluminal locomotion. In *2016 6th IEEE International Conference on Biomedical Robotics and Biomechatronics (BioRob)*, 254–259, 10.1109/BIOROB.2016.7523634 (2016).

[53] Liu Y (2017). Haustral loop extraction for ct colonography using geodesics. International journal of computer assisted radiology and surgery.

[54] Colonoscope training model, kyoto kagaku co., ltd, fushimi-ku kyoto, japan, https://www.kyotokagaku.com/products/detail01/m40.html (Accessed: 22-03-2019).

