# Supplementary material for: A Soft Pneumatic Inchworm Double balloon (SPID) for colonoscopy
Source: Sci Rep. 2019 Jul 31;9:11109. doi: 10.1038/s41598-019-47320-3 (PMC6668406; doi:10.1038/s41598-019-47320-3)
Supplement: Supplementary file 1 — Supplementary materials [file 41598_2019_47320_MOESM1_ESM.pdf]

A Soft Pneumatic Inchworm Double balloon (SPID) for colonoscopy

Luigi Manfredi, Elisabetta Capoccia, Gastone Ciuti, Alfred Cuschieri

Supporting Information

SPID design and fabrication

This section reports the process for creation of the 2 balloons and the SPA, which are constructed and tested separately before assembly. Later, they are glued together to form SPID.

SPA fabrication

The construction process of the SPA consists of 5 steps as depicted in Fig. S1. To achieve an anisotropic behaviour of the SPA for avoidance of lateral expansion during inflation, the outer wall consists of Ecoflex 00-30 (Smooth-on-Inc.TM, PA, USA) reinforced with cotton threads. All the internal walls of the chambers are made of Ecoflex 00-30. Three silicon tubes (outer and inner diameter of 2.6 mm and 1.6 mm respectively) are connected to the base of the SPA and used for activation. The soft polymer (SP) is a composite of Ecoflex 00-30 part A and B mixed in 1:1 ratio. The steps needed to construct the module are shown in Fig. S1. as follows:
Step-1) bottom section of the moulder is assembled and the SP is poured in it, degassed by vacuuming at 0 atm for 10 minutes.
Step-2) insertion of the top section of the moulder, and then cured at 100° for 30 minutes.
Step 3) bottom and external parts of the mould are removed, while the top parts are left inside the cast to keep it rigid. Threads are wrapped around the cast with a gap of 1 mm.
Step 4) bottom section and additional external parts of the mould are assembled, and the SP is poured to form an external layer to protect and fix the cotton threads, forming an anisotropic polymer structure. The cast is again cured at 100° for 30 minutes.
Step 5) the cast is removed and the bottom reinforced sections made of VeroClear-RGB10 polymer together with 3 silicon tubes are glued using Sil-Poxy® Silicone Adhesive.
Quality check of the actuator consists of visual inspection and air leakage testing.

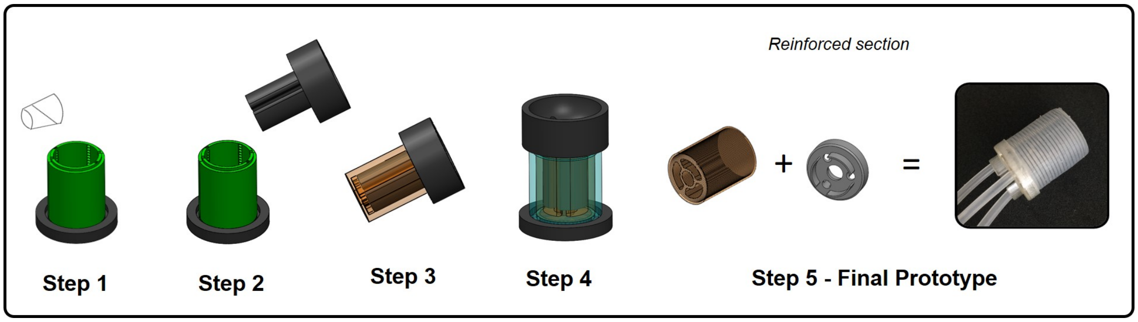

Figure S 1. SPA construction steps.

Balloon fabrication

Construction process of two balloons is similar and includes an internal part made of VeroClear-RGB10 polymer and an external thin layer of Ecoflex 00-30. The internal 3D printed parts are shown in Fig. S2, where IP_1 (internal part 1) relates to the distal balloon, and IP_2 (internal part 2) to the proximal balloon. This frame has an internal silicon tube connector for the balloon activation. The steps needed to construct the balloons are shown in Fig. S2 as follows:
Step 1: bottom section of the moulder is assembled and the SP is poured in it and degassed by vacuuming at 0 atm for 10 minutes.
Step 2: insertion of the top section of moulder.
Step 3: cast is cured at 100° for 30 minutes and then removed from the mould.
Step 4: the top and bottom lips of the cast are folded and glued on top of the IP_1 and IP_2 together with a silicon tube using Sil-Poxy® Silicone Adhesive and left to cure for at least 1 hour.
When both balloons are constructed, visual inspection and air leakage testing is used for quality assessment of the final product.

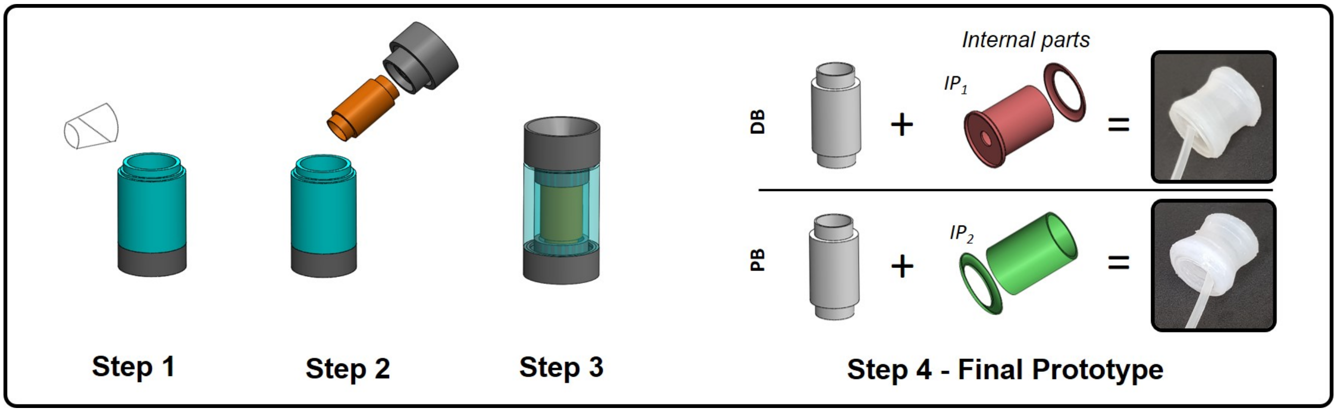

Figure S 2. Balloons construction steps.

SPA and Balloons gluing

The silicon tube for the activation of the distal balloon is inserted inside the SPA after lubrication (Vaseline) to reduce friction in the internal cavity during the locomotion. Then the 3 tubes used for the activation of the SPA are inserted through the proximal balloon and the SPA then glued to the distal and front balloons using Sil-Poxy® Silicone Adhesive and left to cure.

Colon phantom fabrication

The design of the colon phantom is made by using 15 3D printed support rings with the result shown in Fig. S3. A transparent thin plastic film (15 μm thickness) is wrapped around each couple of consecutive rings with lubricant inside to minimise friction. Elastic bands are fixed externally to the plastic film by using Sil-Poxy® Silicone Adhesive to avoid movements.

Experiments

SPA: Blocking force

SPA blocking force experiments were performed by using an Instron® 5564 dual column connected to the tip of the SPA. The 3 chambers were activated simultaneously by controlling the volume of air with 3 piston-cylinders, with an internal volume of 10 mL. Each experiment was performed 5 times in quasi static configuration. The force produced by the SPA when not activated was investigated by measuring the stress strain graphs by using an Instron® 5564 dual column connected to the tip of the SPA. The speed at which the test was run was 1 mm/s with a maximal stroke of 30mm (150 % of its original length).

SPID locomotion

The locomotion experiments were performed by manually controlling the activation of the distal and proximal balloons, together with the SPA. The activation was done by using piston-cylinders to control the volume of air inflated. The piston-cylinder used to activate the balloon has a volume of 100 mL, whereas the 3 piston-cylinders to activate the SPA have a volume of 10 mL each. SPID as well as the silicon tubes were lubricated (Vaseline) to reduce any friction during locomotion.

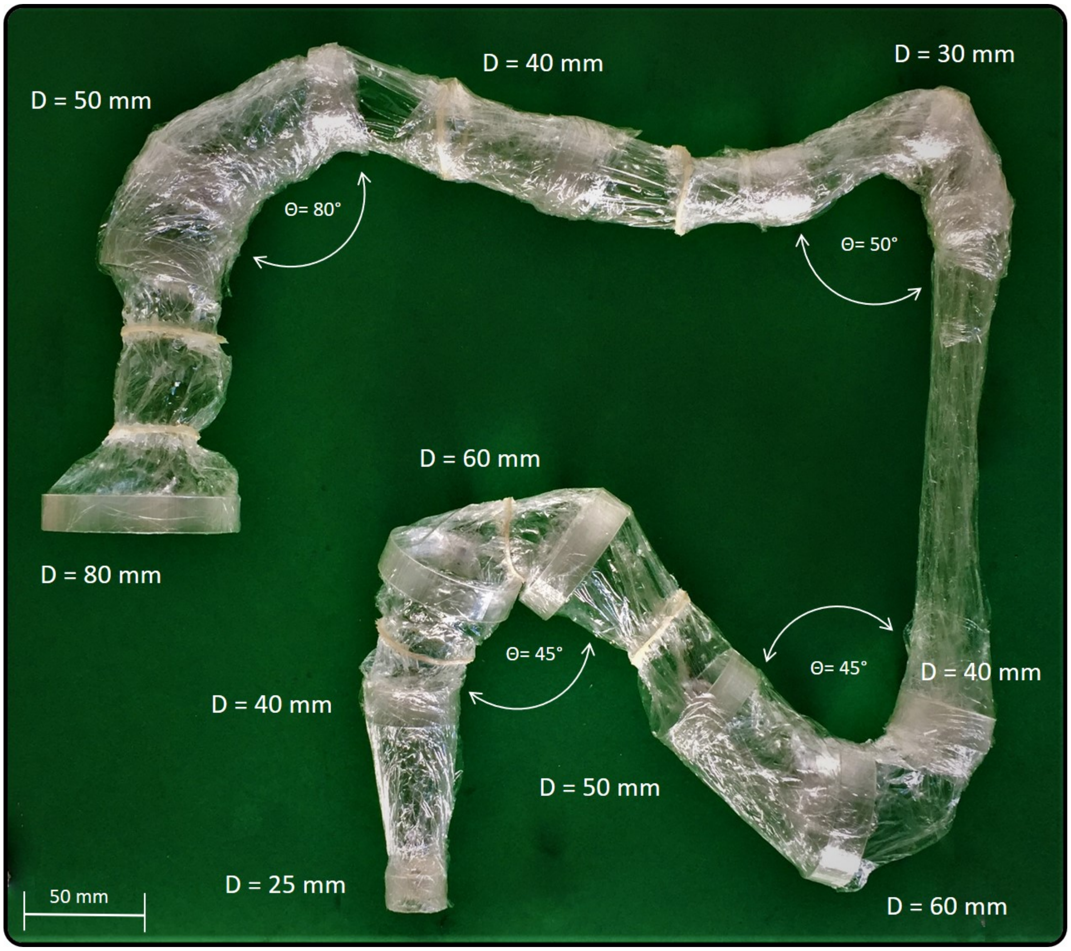

Figure S 3. Colon phantom used for the SPID locomotion experiments. The internal diameters range from 25 mm up to 80 mm, and flexure angles from 45° to 80°.
